# Effect of temperature on fatty acid metabolism in skeletal muscle mitochondria of untrained and endurance-trained rats

**DOI:** 10.1371/journal.pone.0189456

**Published:** 2017-12-12

**Authors:** Jerzy A. Zoladz, Agnieszka Koziel, Izabela Broniarek, Andrzej M. Woyda-Ploszczyca, Karolina Ogrodna, Joanna Majerczak, Jan Celichowski, Zbigniew Szkutnik, Wieslawa Jarmuszkiewicz

**Affiliations:** 1 Department of Muscle Physiology, Faculty of Rehabilitation, University School of Physical Education, Krakow, Poland; 2 Department of Bioenergetics, Adam Mickiewicz University, Poznan, Poland; 3 Department of Neurobiology, University School of Physical Education, Poznan, Poland; 4 Faculty of Applied Mathematics, AGH University of Science and Technology, Krakow, Poland; University of Birmingham, UNITED KINGDOM

## Abstract

We studied the effects of various assay temperatures, representing hypothermia (25°C), normothermia (35°C), and hyperthermia (42°C), on the oxidation of lipid-derived fuels in rat skeletal muscle mitochondria of untrained and endurance-trained rats. Adult 4-month-old male Wistar rats were assigned to a training group (rats trained on a treadmill for 8 weeks) or a sedentary control group. In skeletal muscle mitochondria of both control and trained rats, an increase in the assay temperature from 25°C to 42°C was accompanied by a consistent increase in the oxidation of palmitoylcarnitine and glycerol-3-phosphate. Moreover, endurance training increased mitochondrial capacity to oxidize the lipid-derived fuels at all studied temperatures. The endurance training-induced increase in mitochondrial capacity to oxidize fatty acids was accompanied by an enhancement of mitochondrial biogenesis, as shown by the elevated expression levels of Nrf2, PGC1α, and mitochondrial marker and by the elevated expression levels of mitochondrial proteins involved in fatty acid metabolism, such as fatty acid transporter CD36, carnitine palmitoyltransferase 1A (CPT1A), and acyl-CoA dehydrogenase (ACADS). We conclude that hyperthermia enhances but hypothermia attenuates the rate of the oxidation of fatty acids and glycerol-3-phosphate in rat skeletal muscle mitochondria isolated from both untrained and trained rats. Moreover, our results indicate that endurance training up-regulates mitochondrial biogenesis markers, lipid-sustained oxidative capacity, and CD36 and CPT1A proteins involved in fatty acid transport, possibly via PGC1α and Nrf2 signaling pathways.

## Introduction

Fatty acids are a vital source of energy for mammalian skeletal muscles both when they are at rest and particularly during sustained physical exercise [1–3]. It is well established that the maximal rate of fatty acid oxidation is higher in endurance-trained muscles than in untrained muscles [4–6]. However, the underlying mechanism controlling the maximal rate of fatty acid oxidation in the working muscle remains unclear (for a review, see [3, 6–8]). Endurance training-induced enhancement of the maximal rate of fatty acid oxidation increases exercise tolerance by slowing the depletion of muscle carbohydrates [4, 9]. It has been postulated that training-induced increases in the oxidation of fatty acids by skeletal muscles are caused by the intensification of mitochondrial biogenesis and the resulting increase in the level of fatty acid oxidation enzymes (for a review, see [6, 9, 10]). However, there is a growing body of evidence that the training-induced enhancement of the maximal rate of fatty acid oxidation in skeletal muscles is governed by the up-regulation of fatty acid transporters [7, 8, 11]. It has been proposed that CD36, a fatty acid transporter, may play a key role in the control of the transport of fatty acids into muscle cells and their oxidation at rest and during exercise, as well as in muscle fuel selection and endurance exercise performance [10, 12].

Surprisingly, little attention has been paid to the role of muscle temperature as a potential factor in the regulation of the rate of fatty acid oxidation in skeletal muscles. It is well established that thermal stress can change the proportion of fatty acids and carbohydrates oxidized during exercise, as originally shown by Fink *et al*. [13] and by a series of experiments by Febbraio *et al*. (for a review, see [14]). Namely, it has been reported that exercise in heat seems to increase carbohydrate utilization. However, to the best of our knowledge, no study has been conducted to determine the effect of various temperatures on skeletal muscle mitochondria capacity to oxidize fatty acids. Moreover, no study has been conducted to evaluate the effect of endurance training on the mammalian mitochondrial capacity to oxidize fatty acids under various survivable thermal conditions such as hypothermia, normothermia and hyperthermia (for ref. see [15]. Therefore, the aim of our study is to elucidate (i) the effect of eight weeks of running endurance training on the rate of oxidation of palmitoylcarnitine and glycerol-3-phosphate in mitochondria isolated from rat hind limb skeletal muscles studied at various assay temperatures, 25°C, 35°C, and 42°C, representing hypothermia, normothermia and hyperthermia, respectively, and (ii) the effect of the endurance training on the expression of mitochondrial biogenesis markers (peroxisome proliferator-activated receptor gamma coactivator 1α, PGC1α and nuclear factor E2-related factor 2, Nrf2), a muscle fatty acid transporter CD36 (CD36), and other mitochondrial proteins involved in fatty acid metabolism, i.e., carnitine palmitoyltransferase 1A (CPT1A) and acyl-CoA dehydrogenase (ACADS) in skeletal muscles.

## Materials and methods

### Animals

The study was performed on 24 adult 4-month-old male Wistar rats. Animals were randomly assigned to either a training group (*n* = 12) or a sedentary control group (*n* = 12). During the experiment, the animals were kept in standard laboratory cages in a room with a 12 h/12 h light/dark cycle, controlled temperature (22 ± 2°C) and humidity (55 ± 10%). All rats had unrestricted access to standard rat feed and tap water.

Experimental protocols involving animals, their surgery and care were approved by the Local Ethics Committee on Animal Experimentation in Poznan, Poland (Permit Number: 15/2013) and were in compliance with the guidelines of the European Community Council Directive 2010/63/UE of 22 September 2010 on the protection of animals used for scientific purposes. Animals were sacrificed, and all efforts were made to minimize suffering.

### Rat endurance training

The eight-week training was performed at 22 ± 2°C, 5 times per week (Monday-Friday) on a standard treadmill for small rodents (Exer 3/6 M Treadmill, Columbus Instruments, Columbus, OH, USA) as described previously [16]. The running belt was placed horizontally with a 0° inclination. In the first week of the training, the rats were familiarized with running on the treadmill at various velocities (20–30 m x min^-1^) during 20–30 min running sessions. At the end of the first week, the duration of a training session was increased to approximately 40 min. In the first two weeks of training, the basal running velocity was set at 30 m x min^-1^, but every 10 min it was increased to 40 m x min^-1^ for approximately 20 s. From the fifth week of training, the duration of the training sessions was extended to approximately 60 min. The basal running velocity at this stage of training was set to 30 m x min^-1^, and approximately every 10 min the running velocity was increased up to 40 m x min^-1^. The duration of the higher speed was gradually increased from 20 s in the 6th week to approximately 40 s in the final week of training. At the end of the training period, 22–24 h after completing the last training session, exercising and sedentary rats were sacrificed by decapitation. All efforts were made to minimize suffering.

### Skeletal muscle homogenate and mitochondria preparation

Immediately after decapitation of the animals (both control and trained rats) hind limbs were rapidly removed at the level of the hip joints in order to dissect all major hind limb locomotor muscles (the gastrocnemius, soleus, and quadriceps muscles). In order to obtain sufficient mass of muscle tissue to collect adequate amount of mitochondrial fraction the obtained muscles from shank and thigh were used as a mixed muscle sample to obtain muscle homogenates and mitochondria fractions for further measurements as previously described [15, 17].

We have assessed purity of mitochondrial fractions by detection of peroxisome contamination (S1 Fig). In contrast to muscle homogenates, mitochondrial fractions probed with antibody to peroxisome membrane marker (PMP70) displayed no signal, indicating the absence of peroxisome membrane contamination in mitochondrial preparations.

### Protein concentration determination

Muscle homogenate and mitochondrial protein concentration was determined using the Bradford method with BSA as a standard.

### Measurements of mitochondrial respiration

Measurements were performed in isolated mitochondria at various temperatures (25°C, 35°C and 42°C) as described previously [15, 17]. Our choice of the assay temperatures was based on the previous studies showing that muscle temperature at rest in rats [18] and humans [19, 20] amounts to ~35°C-36°C. Moreover, it has been reported that rats can survive a decrease in their core temperature to ~15°C and its increase to ~43°C [21, 22]. Similar values of the highest and the lowest survivable core temperatures have been reported for humans [23, 24]. Therefore, the assay temperatures applied in our study to mimic hypothermia (25°C), normothermia (35°C), and hyperthermia (42°C) represent the range of survivable temperatures for rodents and humans.

Oxygen uptake was determined polarographically using a Hansatech oxygen electrode in 0.7 ml of respiration medium (225 mM mannitol, 75 mM sucrose, 10 mM KCl, 5 mM KH_2_PO_4_, 0.5 mM EDTA, 0.5 mM EGTA, 0.05% BSA, 10 mM Tris-HCl, pH 7.2), with 0.25 mg of mitochondrial protein (0.36 mg x ml^-1^). To chelate the endogenous free fatty acids in mitochondrial preparations (especially in mitochondria from trained rats), 0.05% BSA was always present in the respiration medium.

Mitochondrial oxidation of palmitoylcarnitine and glycerol-3-phosphate were measured with 0.5 mM palmitoyl-DL-carnitine or 5 mM glycerol-3-phosphate as respiratory substrates, respectively. Respiratory rates were measured in the absence (state 4, non-phosphorylating respiration) or presence of 150 μM ADP (state 3, phosphorylating respiration).

### Determination of protein levels through immunoblotting

RIPA buffer (150 mM NaCl, 1% Triton X-100, 0.5% Na deoxycholate, 0.1% SDS, 50 mM Tris, pH 8.0) was used to lyse the rat muscle homogenates. The muscle homogenates and mitochondrial fractions were isolated in the presence of protease inhibitors. The proteins were separated on 8%, 10% or 12% SDS-PAGE gels. The following primary antibodies were used: anti-PGC1α (Abcam), anti-Nrf2 (Abcam), anti-CD36 (Santa Cruz Biotechnology), anti-CPT1A (Abcam), anti-ACADS (Abcam), anti-cytochrome *c* subunit II (COXII) (Abcam), anti-β actin (Calbiochem), anti-glyceraldehyde-3-phosphate dehydrogenase (GAPDH, 37 kDa) (Abcam), and anti-mitochondrial marker (MTC02) (Abcam). Appropriate horseradish peroxidase-conjugated secondary antibodies were used. The expression levels of β-actin or GAPDH (for the homogenate fractions) and COXII or mitochondrial marker (for the mitochondrial fractions) were used as loading normalization controls. Protein bands were visualized using the Amersham ECL system and digitally quantified using the GeneTools 4.03 software package.

### Statistical analysis

The results are presented as the mean ± SD (*n* = 6) obtained from six independent muscle homogenate or mitochondrial preparations. Each isolation was performed from one control or trained animal. Functional measurements and immunodetections were performed in triplicate. The experimental data concerning the effect of temperature and/or training on oxidation of lipid-derived fuels were analyzed with mixed repeated-measures ANOVA, with one within-subjects factor (temperature) and one between-subjects factor (control/training). Differences in reactions to training, measured at different temperatures, would result in a non-zero interaction between the factors. Both univariate and multivariate tests for repeated measures in all studied variables resulted in non-significant interactions, however. The significance of differences between the levels of the studied variables (three training-control comparisons at various temperatures for each variable) were examined using planned comparisons. The obtained *P* values were corrected using the Holm–Bonferroni method. The effect of endurance training on the expression level of muscle proteins was analyzed using unpaired *t-*test. Differences were considered to be statistically significant if *P* < 0.05.

## Results

### The effect of endurance training on mitochondrial oxidation of lipid-derived fuels at various temperatures

**Fig 1:**
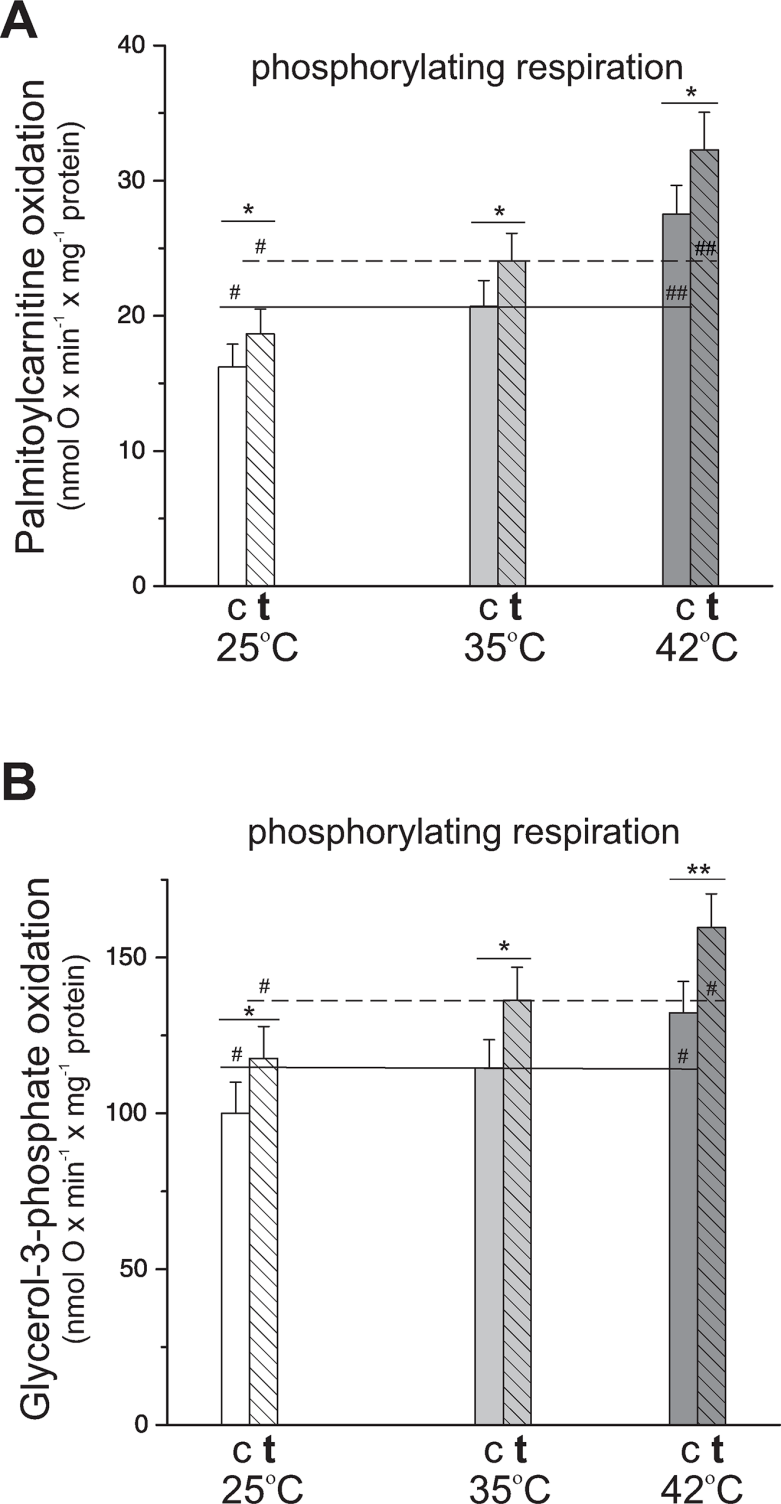
**The influence of endurance training on mitochondrial oxidation of palmitoylcarnitine (A) and glycerol-3-phosphate (B) at 25**°**C, 35**°**C and 42**°**C.** Respiratory rates were measured in mitochondria isolated from control (c) and trained (**t**) rats in the presence of 150 μM ADP (state 3, phosphorylating respiration) with 0.5 mM palmitoyl-DL-carnitine (**A**) or 5 mM glycerol-3-phosphate (**B**). Mean values (± SD) for six mitochondria preparations from six different animals from each group (*n* = 6) are shown. ^###^*P* < 0.001, ^##^*P* < 0.01, ^#^*P* < 0.05 vs value obtained at 35°C for a given group of animals, i.e., within control rats or trained rats. ***P < 0.001, **P < 0.01, *P < 0.05 vs. value obtained for control rats for a given assay temperature.

In the present study, we shown for the first time that, in isolated mitochondria from both untrained and trained rat hind limb skeletal muscles, an increase in the assay temperature from 25°C to 42°C resulted in augmentation of the maximal oxidation of palmitoylcarnitine and glycerol-3-phosphate under phosphorylating (state 3) conditions (Fig 1). For each assay temperature, the oxidation of the lipid-derived fuels was significantly higher in skeletal muscle mitochondria isolated from endurance-trained rats compared to that isolated from control animals. Interestingly, the endurance training-induced increase in oxygen consumption with palmitoylcarnitine (by 16%-18%) and glycerol-3-phosphate (by 18%-20%) was similar along with an increase in the assay temperature from 25°C to 42°C (i.e., at 25°C, 35°C, and 42°C).

The non-phosphorylating respiration (in the absence of ADP) and respiratory control ratio were studied for the three tested temperatures in mitochondria isolated from the skeletal muscles of trained and control rats using glycerol-3-phosphate as a respiratory substrate (Table 1). In both types of mitochondria, beside the increase in phosphorylating respiration (state 3), an elevation of non-phosphorylating respiration (state 4) was observed with increasing temperature. For a given assay temperature, a significant increase in phosphorylating respiration was accompanied by a significant decrease in non-phosphorylating respiration in muscle mitochondria from trained rats compared to those of the controls. In both types of mitochondria, the respiratory control ratio decreased significantly with the increasing assay temperature, indicating a temperature-induced mitochondrial uncoupling. Independent of temperature, the respiratory control ratio was considerably higher in mitochondria from the muscles of trained rats than from the muscles of control rats, indicating that endurance training leads to less uncoupling in muscle mitochondria.

**Table 1:** Respiratory rates and coupling parameters in control and trained rat skeletal muscle mitochondria at 25°C, 35°C and 42°C.

	Control rat mitochondria			Trained rat mitochondria		
Glycerol-3-phosphate	25°C	35°C	42°C	25°C	35°C	42°C
State 3 rate	# 100 ± 10	115 ± 9	# 132 ± 10	# 118 ± 10 *	136 ± 11 *	# 160 ± 11 **
State 4 rate	### 52.5 ± 4.1	69.5 ± 4.6	### 104 ± 6.9	### 43.8 ± 3.6 **	60.7 ± 4.5 *	### 91.5 ± 6.3 **
RCR	# 1.91 ± 0.19	1.64 ± 0.19	## 1.27 ± 0.13	## 2.69 ± 0.24 ***	2.24 ± 0.23 **	### 1.75 ± 0.15 ***

Respiratory rates were measured in the absence (state 4, non-phosphorylating respiration following phosphorylating respiration) or presence (state 3, phosphorylating respiration) of 150 μM ADP with 5 mM glycerol-3-phosphate. The respiratory rates (in nmol O x min^-1^ x mg^-1^ protein) of state 3 and state 4 as well as corresponding respiratory control ratios (RCR) are presented. The RCR is equal to the ratio of state 3 to state 4 respiration. Mean values (± SD) for six different mitochondria preparations (*n* = 6) are shown.

^###^*P* < 0.001

^##^*P* < 0.01

^#^*P* < 0.05, comparison vs. value obtained at 35°C for a given group.

***P < 0.001

**P < 0.01

*P < 0.05, comparison vs. value obtained for control rats for a given temperature.

### The effect of endurance training on skeletal muscle mitochondrial biogenesis

**Fig 2:**
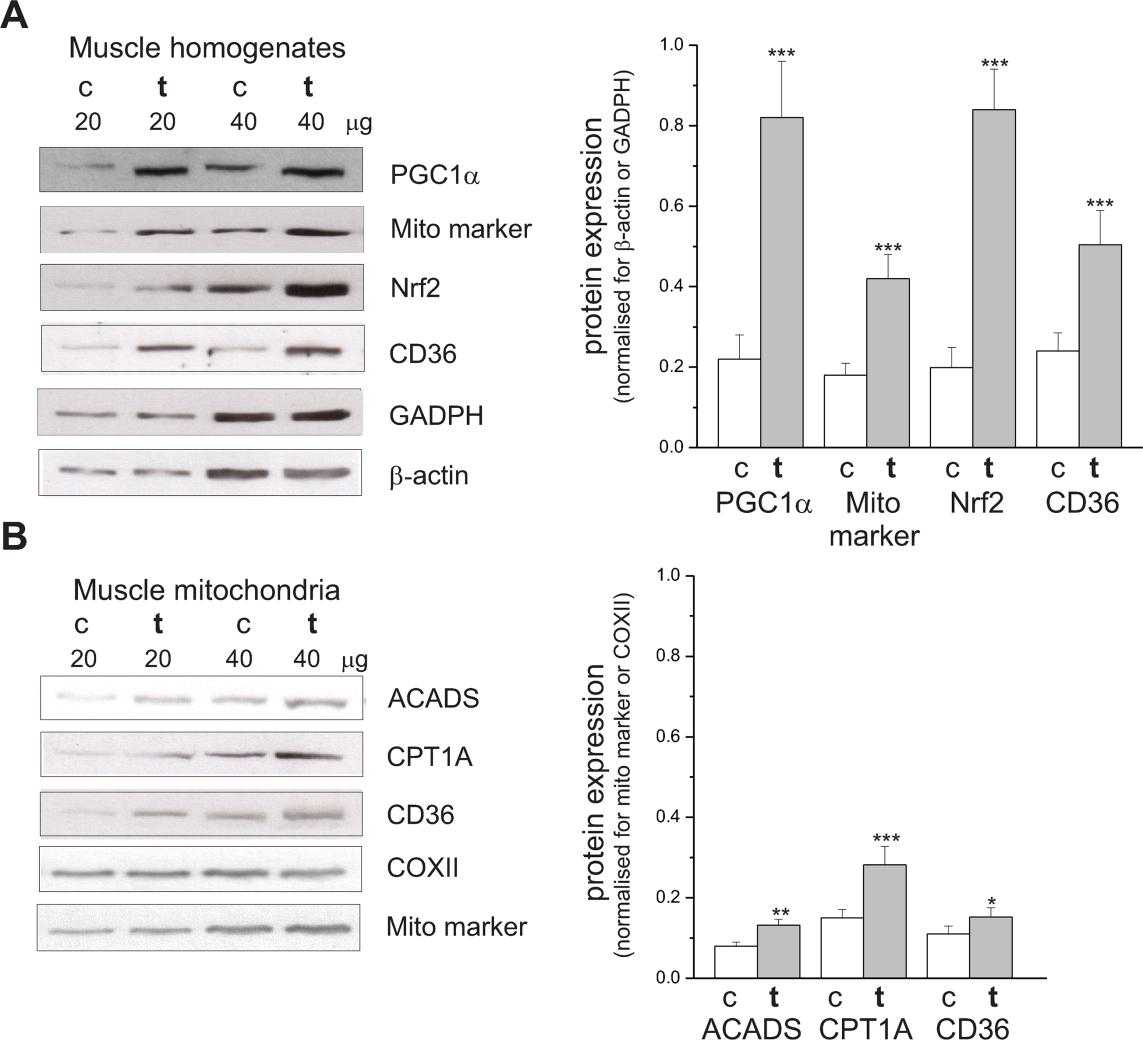
**Determination of protein levels in skeletal muscle homogenates (A) and mitochondria (B) from control (**c**) and trained (t) rats. A**, Representative Western blots and analyses of the protein expression of PGC1α, mitochondrial marker (Mito marker), Nrf2, CD36, β-actin and GADPH. **B**, Representative Western blots and analyses of the protein expression of ACADS, CPT1A, CD36, COXII, (Mito marker). Expression levels were normalized for β actin or GADPH (**A**, *right panel*) and mito marker or COXII (**B**, *right panel*). The data (± SD, *n* = 6) is from six independent homogenate or mitochondrial preparations from six different animals from each group. ***P < 0.001, **P < 0.01, *P < 0.05 vs. value obtained for control rats.

The eight-week endurance training program applied in this study resulted in a significant increase in mitochondrial biogenesis in the locomotor hind limb muscles of trained rats. A significant augmentation in the protein expression level of mitochondrial biogenesis markers, such as the transcriptional coactivator that regulates genes involved in energy metabolism PGC1α (~3.7-fold increase), mitochondrial marker (~2.1-fold increase), and the transcription factor Nrf2 (~4-fold increase), was observed in the skeletal muscle homogenates of trained rats compared to control animals (Fig 2A).

### The effect of endurance training on protein expression of CD36, CPT1A, and ACADS

Interestingly, in addition to the increase in the protein level of Nrf2 (Fig 2A), which reflects the increase in mitochondrial biogenesis and an enhancement of fatty acid oxidation [25], a significant augmentation of the level of a fatty-acid transport protein, CD36 (~2.1-fold increase), was observed in the muscle homogenates of trained rats (Fig 2A). Moreover, in our study, in mitochondria isolated from the skeletal muscles of trained rats, the expression level of CD36 was enhanced significantly (by ~38%) compared to that in control animals (Fig 2B). Moreover, significantly increased levels of other mitochondrial proteins involved in fatty acid metabolism, i.e., CPT1A (by ~90%) and ACADS (by ~65%) were observed in mitochondria isolated from trained rats (Fig 2B).

## Discussion

In the present study, we have shown that (i) hyperthermia enhances but hypothermia attenuates the rate of the oxidation of fatty acids and glycerol-3-phosphate in rat skeletal muscle mitochondria isolated from both untrained and trained rats, (ii) endurance training up-regulates mitochondrial lipid-sustained oxidative capacity in all studied temperatures, and (iii) endurance training enhances muscle mitochondrial biogenesis markers and CD36 and CPT1A proteins involved in fatty acid transport, possibly via PGC1α and Nrf2 signaling pathways.

### Assay temperature and mitochondrial fatty acid oxidation

The present study is the first to examine the effect of various temperatures in the range of 25°C-42°C on the capacity of isolated skeletal muscle mitochondria to oxidize fatty acids (Fig 1A). Therefore, we cannot directly relate our results to previous studies. Our study clearly shows that an increase in the assay temperature from the state of hypothermia (25°C) to hyperthermia (42°C), thus in the range of temperatures that humans can survive [15], gradually increases the rate of fatty acid oxidation by isolated skeletal muscle mitochondria. Therefore, the present study is the first to show that hyperthermia is not a limiting factor for mitochondrial capacity to oxidize fatty acids and that endurance training performed at room temperature i.e., ~22°C, enhances mitochondrial capacity for fatty acid oxidation at all studied temperatures (25°C-42°C). Our findings concerning the impact of assay temperature on the mitochondrial capacity for fatty acid oxidation are in accordance with the early study by Brooks *et al*. (1971) [18], who have originally reported that an increase in assay temperature between 25°C-45°C enhances mitochondrial state 3 respiration with pyruvate and malate as respiratory substrates. We consider that the temperature-induced increase in mitochondrial fatty acid oxidation observed in our study represents qualitative temperature-induced changes in the muscle mitochondria resulting mainly from the temperature-related enhancement of mitochondrial enzyme activities.

Our results obtained under *in vitro* conditions cannot be simply transferred into *in vivo* conditions, in which the regulation of fatty acid transport into mitochondria is much more complex and involves several possible limiting steps (for an overview see, *e*.*g*., [26]) not present in our experimental model. Nevertheless, our study suggests that the exercise-induced hyperthermia that occurs during high-intensity sustained exercise [19] could not limit and even could potentiate muscle mitochondrial capacity for fatty acid oxidation. Whereas hypothermia that can be induced for example by body exposure to low ambient temperatures could decrease the maximal rate of muscle mitochondrial fatty acid oxidation. It could be one of the factors that limits exercise performance at very low ambient temperatures. However, the extent to which our *in vitro* results relate to mitochondrial function *in vivo* remains to be determined.

### Endurance training and mitochondrial oxidation of lipid-derived fuels

Our results show that oxidation of reducing substrates originating from lipid catabolism, i.e., fatty acid β-oxidation and glycerol-3-phosphate oxidation, in skeletal muscle mitochondria of trained rats is greater than that of untrained control animals (Fig 1). This observation is in agreement with previous studies showing that endurance training increases the rate of mitochondrial fatty acid oxidation in skeletal muscles [12, 27, 28]. However, it is worth to underline that in the present study, we have shown for the first time that the training-induced increase in the maximal rate of muscle mitochondrial fatty acid oxidation was similar (16%-18%) at all studied temperatures (Fig 1A). This observation indicates that the mechanism responsible for the training-induced enhancement of mitochondrial capacity for fatty acid oxidation is temperature-independent. This property might explain the beneficial effect of endurance training performed under thermoneutral conditions for enhancement of physical performance at high and low temperatures.

A considerable increase in the protein expression level of mitochondrial biogenesis markers (PGC1α, mitochondrial marker, and Nrf2), was observed in the skeletal muscle homogenates of trained rats compared to control animals (Fig 2). We have recently reported that the eight-week endurance training of 4-month-old male Wistar rats resulted in an enhancement of mitochondrial biogenesis and oxidative phosphorylation (OXPHOS) capacity, as shown by a significant increase in the maximal cytochrome *c* oxidase (COX) (~65%-75%) and citrate synthase (CS) (~60%-70%) activities, as well as the levels of these proteins in the muscles of trained rats [17]. Maximal COX activity is considered the best measure of OXPHOS capacity in skeletal muscles [29, 30]. The results of other groups also show that prolonged endurance training increases muscle mitochondrial biogenesis [31–34]. In the present study, the endurance training-induced increase in OXPHOS activity was directly confirmed by a significant increase in oxidation of palmitoylcarnitine and glycerol-3-phosphate in skeletal muscle mitochondria from trained rats compared to control animals (Fig 1). The increase in OXPHOS activity leads to the improvement of muscle metabolic stability during exercise of given power output, resulting in increased resistance to muscle fatigue [30, 33, 35, 36].

In skeletal muscle mitochondria of untrained and endurance-trained rats, during glycerol-3-phospate oxidation with the increasing assay temperature (from 25°C to 42°C), an elevation of non-phosphorylating respiration (state 4) was observed beside the increase in phosphorylating respiration (state 3) (Table 1). However, in mitochondria from both control and trained rats, the resulting respiratory control ratio decreased significantly with the increasing assay temperature, indicating a temperature-induced mitochondrial uncoupling. Independent of temperature, the respiratory control ratio was considerably higher in mitochondria from the muscles of trained rats than from the muscles of control rats, indicating that endurance training attenuates high temperature-elevated mitochondrial uncoupling. The decrease in the high temperature-elevated mitochondrial uncoupling indicates a higher need for an efficient ATP synthesis in skeletal muscle mitochondria of trained rats compared to those of untrained animals and could be beneficial for muscle performance by decreasing oxygen cost of work at a given power output (the V’O_2_/power output ratio). These results are consistent with our previous measurements performed with substrates of the Krebs cycle (succinate and malate) in rat skeletal muscle mitochondria isolated from control and trained rats [17].

### Endurance training and expression level of CD36, CPT1A, and ACADS

Our results indicate that under phosphorylating conditions, endurance training may augment the temperature-induced increase in maximal oxidation of reducing substrates originating from lipid catabolism. The underlying physiological mechanism responsible for the endurance training-induced increase in fatty acid oxidation is not completely understood. Two factors are currently considered to explain this muscle adaptive response: (i) the training-induced enhancement of mitochondrial oxidative capacity [6, 9, 10], and (ii) the training-induced enhancement of fatty acid transporters, especially CD36 [10, 12, 37] and CPT1-dependent mitochondrial transport [6]. In our study, the training-induced increase in mitochondrial fatty acid oxidation was accompanied by an increase in expression level of and proteins considered to determine the rate of mitochondrial fatty acid oxidation, i.e., mitochondrial biogenesis markers (PGC1α and Nrf2) and proteins involved in fatty acid metabolism (CD36, CTP1A, and ACADS) (Fig 2).

For a long time, mitochondrial biogenesis and the resulting increase in the level of enzymes catalyzing fatty acid oxidation was considered as the main mechanism of training-induced enhancement of fatty acid oxidation [6, 9, 10]. However, this concept has been challenged by recent studies performed with the CD36 knockout mice model, which show that the selective training-induced enhancement of fatty acid transporters, especially the CD36, and not the increase of mitochondrial oxidative capacity is the key factor responsible for the training-induced enhancement of fatty acid oxidation in the endurance trained skeletal muscles [10, 12].

Our finding of the training-induced increase in CD36 in the trained skeletal muscles (Fig 2A) is in agreement with a previous studies showing a similar muscle response to training [10, 12, 38]. Moreover, in our study, in mitochondria isolated from the skeletal muscles of trained rats, the expression level of CD36 was enhanced significantly (Fig 2B). Recently, it has been reported that in skeletal muscle, CD36 is located on the outer mitochondrial membrane, upstream of long-chain acyl-CoA synthetase, and regulates palmitate oxidation [39]. Moreover, significantly increased levels of other mitochondrial proteins involved in fatty acid metabolism, i.e., CPT1A, which catalyzes the transfer of the acyl group of a long-chain fatty acyl-CoA from coenzyme A to carnitine and ACADS, which catalyzes the initial step of fatty acid β-oxidation, were observed in mitochondria isolated from trained rats (Fig 2B). Thus, endurance training enhanced the CPT1 system, which controls the entry of long-chain fatty acid acyl-CoA into mitochondria and mitochondrial long-chain fatty acid oxidation.

Our results show that the training-induced intensification of mitochondrial biogenesis is accompanied by the increase in expression levels of fatty acid transporter CD36 and other mitochondrial proteins involved in fatty acid metabolism, such as CPT1A and ACADS. This suggests that under physiological conditions, endurance training up-regulates both the fatty acid transporter and muscle mitochondrial oxidative capacity. This observation is in agreement with recent findings showing that PGC1α is a potent activator of mitochondrial biogenesis (for a review, see [40]) and induces the expression of CD36 and stimulates muscle fatty oxidation [10, 41]. It has also been reported that Nrf2 is responsible for regulating the expression of proteins of the mitochondrial electron transport chain [40] and also involved in the regulation of the rate of fatty acid oxidation [25]. Thus, the observed in the present study up-regulation of fatty acid transporters accompanied by the increase in mitochondrial oxidative capacity to oxidize fatty acid suggest that in skeletal muscles under physiological conditions, endurance training up-regulates both of these processes, probably with the involvement of PGC1α and Nrf2 being elevated by the endurance training. This muscle adaptive response might play a key role in the mechanism of the training-induced increase of endurance exercise performance *via* an enhancement of ATP supply from fatty acid oxidation allowing for slowing-down the rate of muscle glycogen depletion, considered as a core factor of muscle fatigue [9, 36, 42, 43].

## Conclusions

In this study, we have demonstrated for the first time that hyperthermia enhances but hypothermia attenuates the oxidation rate of fatty acids and glycerol-3-phosphate in rat skeletal muscle mitochondria isolated from both untrained and trained rats. We postulate that this effect is caused by the temperature-related enhancement of mitochondrial enzyme activities. Furthermore, we have shown that endurance training increases significantly mitochondrial lipid-sustained oxidative capacity at all studied temperatures (20°C, 35°C, and 42°C). Moreover, our results further support the notion that the endurance training-induced up-regulation of mitochondrial lipid-sustained oxidative capacity involves an enhancement of mitochondrial biogenesis and CD36- and CPT1A-dependent fatty acid transport, possibly via PGC1α and Nrf2 signaling pathways.

## Supporting information

S1 Fig Assessment of mitochondrial fraction purity by detection of peroxisome contamination. Muscle homogenates (H) and mitochondrial fractions (M) from control (c) and trained (**t**) rats were probed with antibody to ~70 kDa peroxisome membrane marker (PMP70, Abcam). A representative immunodection is shown.
(PDF)
