# Supplementary material for: Effect of temperature on fatty acid metabolism in skeletal muscle mitochondria of untrained and endurance-trained rats
Source: PLoS One. 2017 Dec 12;12(12):e0189456. doi: 10.1371/journal.pone.0189456 (PMC5726737; doi:10.1371/journal.pone.0189456)

# Supporting information

**S1 Fig. Assessment of mitochondrial fraction purity by detection of peroxisome contamination.** Muscle homogenates (H) and mitochondrial fractions (M) from control (c) and trained (**t**) rats were probed with antibody to ~70 kDa peroxisome membrane marker (PMP70, Abcam). A representative immunodection is shown.

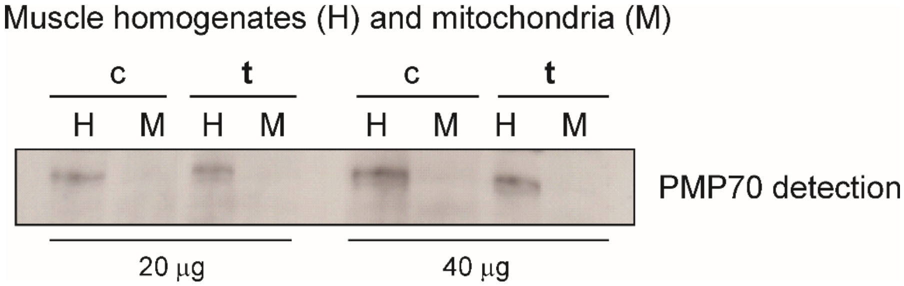

Supplement: S1 Fig — Muscle homogenates (H) and mitochondrial fractions (M) from control (c) and trained (t) rats were probed with antibody to ~70 kDa peroxisome membrane marker (PMP70, Abcam). A representative immunodection is shown. (PDF) [file pone.0189456.s001.pdf]
